# Detection of In Vivo-like Cells by a Biosensor Chip Based on Metamaterials in Terahertz Regime

**DOI:** 10.3390/bios14050230

**Published:** 2024-05-06

**Authors:** Lulu Han, Yuchen Wang, Kanglong Chen, Hengyu Gao, Kexin Xia, Qinggang Ge, Jun Yang, Wei Shi, Cunjun Ruan

**Affiliations:** 1 School of Electronic and Information Engineering, Beihang University, Beijing 100191, China; hanlulu@buaa.edu.cn (L.H.); sharksion@163.com (K.X.); 2 School of Biological Science and Medical Engineering, Beihang University, Beijing 100191, China; by2110205@buaa.edu.cn (Y.W.); 20374379@buaa.edu.cn (H.G.); 3 Department of Neurosurgery, Peking University Third Hospital, Beijing 100191, China; qingganggelin@126.com (Q.G.); yangjbysy@bjmu.edu.cn (J.Y.); 4 Center for Precision Neurosurgery and Oncology of Peking University Health Science Center, Beijing 100191, China; 5 Beijing Key Laboratory for Microwave Sensing and Security Applications, Beihang University, Beijing 100191, China

**Keywords:** terahertz, metamaterials, biosensor, live cell

## Abstract

Early diagnosis of diseases, especially cancer, is critical for effective treatment. The unique properties of terahertz technology have attracted attention in this field. However, current terahertz bio-detection methods face challenges due to differences between the test environment and the actual in vivo conditions. In this study, a novel method is proposed for detecting in vivo-like cells using a biosensor chip composed of metamaterials and a cavity. The cavity has a thickness of ~50 μm. The structure can protect cells from damage and provides a liquid environment like an in vivo state. Through simulation analysis, the metamaterials sensor exhibits a theoretical sensitivity of 0.287 THz/RIU (Refractive Index Unit) with a 50 μm thick analyte. The detection method is experimentally validated using the apoptosis of glioma cells and various cell types. The biosensor investigates the apoptosis of glioma cells under the impact of temozolomide, and the trend of the results was consistent with the Cell Counting Kit-8 method. Furthermore, at a concentration of ~5200 cells/cm^2^, the experimental results demonstrate that the sensor can distinguish between neurons and glioma cells with a resonance frequency difference of approximately 30 GHz. This research has significant potential for detecting glioma cells and offers an alternative approach to in vivo-like cell detection.

## 1. Introduction

Gliomas, primary brain tumors, are thought to originate from the neuroglial stem or progenitor cells [1]. The World Health Organization (WHO) classified them into four malignancy grades, ranging from grade I to IV [1,2,3]. WHO grade I usually spread slowly and have a more benign behavior. WHO grades II and III can spread faster and act more aggressively. WHO grade IV is the most malignant type, also known as glioblastoma (GBM) [3]. The incidence of gliomas generally increases with age, with the highest increase observed in GBM [4]. Therefore, early detection and effective treatment of GBM are urgently needed. Currently, the traditional GBM diagnosis techniques mainly include computed tomography (CT), magnetic resonance imaging (MRI), immunohistochemistry, and hematoxylin and eosin staining (HE) section. Unfortunately, these detection techniques come with the drawbacks of being complex, expensive, poor accuracy, and radiation exposure [5].

Terahertz (THz) detection of GBM has attracted considerable attention in recent years due to its unique properties, such as low ionization and label-free [6,7]. For micro-gliomas, the resonance between the THz signal and the sample is negligible, making identifying characteristic peaks of the spectrum impossible. Metamaterials (MMs), periodic artificial structures that can localize and enhance electromagnetic fields, can be applied to highly sensitive THz biological detection [8]. However, in the THz biosensor application, the state of samples is crucial for tumor identification. Due to the strong water absorption at THz frequencies, THz sensors are typically limited to dry or partially hydrated specimens. For example, Li (2021) [9] used an MMs-based THz biosensor to identify early-stage cervical cancerous tissues. Chen (2023) [10] employed an MMs-based THz biosensor to detect the low-concentration dried GBM cells deposited on the surface of MMs. Zhang (2018) [11] proposed an MMs-based THz biosensor to analyze the apoptosis of dried cancer cells cultured on the surface of MMs. These methods have a common problem: the sample is exposed to a bare environment (e.g., air or nitrogen) during the test, making it difficult to maintain cell viability. They also face challenges such as ensuring sample thickness consistency, reducing the number of sensors required for multiple data sets, preventing MM structure damage during cleaning, and cultivating adherent cells uniformly on the metal pattern of MMs. In conclusion, these studies encounter many problems, such as cell inactivation, poor cell consistency, and high cost. Therefore, it is crucial to develop new methods and technologies for highly sensitive THz detection of living cells as close as possible to an in vivo-like state.

In this study, we propose a method for detecting cells that mimic the in vivo state using a biosensor chip composed of MMs and a cavity. This method employs a high-sensitivity MMs sensor to improve the detection sensitivity and incorporates adherent cells with a liquid environment to simulate the in vivo condition. Additionally, the cavity has a thickness of ~50 μm, which can effectively weaken the strong water absorption of THz waves, preserve the liquid environment, and ensure the uniformity of sample thickness. By applying terahertz time-domain spectroscopy (THz-TDS), we can evaluate the apoptosis of glioma cells, as well as differentiate between neurons and glioma cells. This method offers many advantages, such as higher sensitivity, low absorption liquid test, and consistent sample thickness. Our proposed method provides a new approach to enable cells to closely mimic the in vivo state and has the potential for various medical applications.

## 2. Design

### 2.1. MMs Sensor

**Figure 1:**
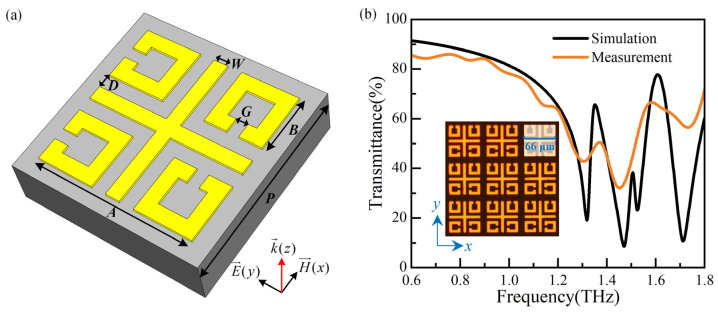
(**a**) The unit cell of the MMs sensor, and (**b**) the simulation and measurement results of the MM sensor.

A high-sensitivity sensor based on MMs is proposed, which consists of four split-ring resonators (SRRs) arranged asymmetrically and a cross, as presented in Figure 1a. The coupling capacitance is increased by using the gap of SRRs and between SRRs and the cross, resulting in improved sensitivity [10]. The metal structure is gold with an electric conductivity of 4.561 × 10^7^ S/m and a thickness of 0.2 μm. The substrate is quartz (lossy) with a dielectric constant *ɛ*_r_ = 3.75 and a thickness of 1000 μm. The geometrical parameters are *P* = 80 μm, *A* = 66 μm, *B* = 24 μm, *D* = 6 μm, *W* = 6 μm, and *G* = 5 μm. Numerical simulations are carried out using CST Microwave Studio (2016). The periodic boundary condition of the unit cell is applied in the *x*- and *y*-directions and open (add space) in the *z*-direction. Electric and magnetic boundary conditions are adopted along *y*- and *x*-directions to simulate the *E_y_*-polarized incident waves propagating along the *z*-direction. The sensor is fabricated using MEMS processing technology, and the photograph of the physical device is shown in the insert of Figure 1b.

The simulation and measurement results of the MMs sensor are shown in Figure 1b. The measurement results exhibit some variations in amplitude within transmission curves, while the overall envelope closely agrees with simulation results, except for a resonance dip at around 1.5 THz. This dip remains elusive due to its high Q-factor and the limited scanning time during measurement imposed by the substrate thickness [12].

While the sensor has already been discussed in [10], this paper introduces a novel detection method for in vivo-like cells, addressing the problem of biological inactivation encountered during testing in the reference mentioned above. This method can be extended to cancer detection research with improved sensitivity [13,14]. The resonance frequency (*f*) of the biosensor can be expressed as *f*∝1/(2*dε*_eff_^1/2^), where the geometric parameters of the biosensor primarily determine the value of *d*, while the effective dielectric constant of the environment is represented by *ε*_eff_. Cells on the biosensor’s surface can cause a change in *ε*_eff_, resulting in a shift of the resonance frequency in the MMs. This study uses a resonance frequency of ~1.32 THz (test result: ~1.31 THz), different from previous work.

**Figure 2:**
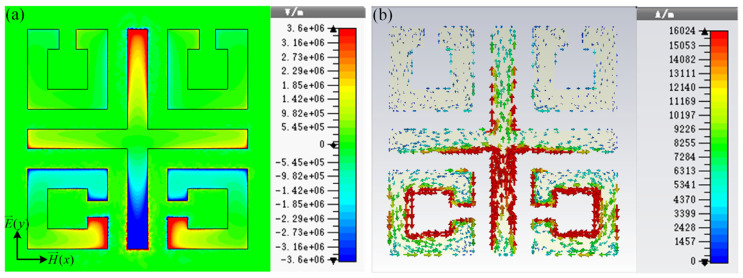
(**a**) Normal electric field and (**b**) surface current distributions of the sensor at 1.32 THz.

To study the physical mechanism of the resonance of the high-sensitivity sensor, the normal electric field and surface current distributions of the sensor at 1.32 THz are presented in Figure 2. As two SRRs located above the structure are perpendicular to the incident electric field, the coupling of the left and right SRR to the incident electric field is very weak. The electric field is mainly concentrated around the gap of the open ring below the sensor and at both ends of the *y*-axis of the cross wire, resulting in a multipole-like dipole resonance. The dipole resonance causes the main losses of the MM structure.

### 2.2. Integration of Biosensor Chip

**Figure 3:**
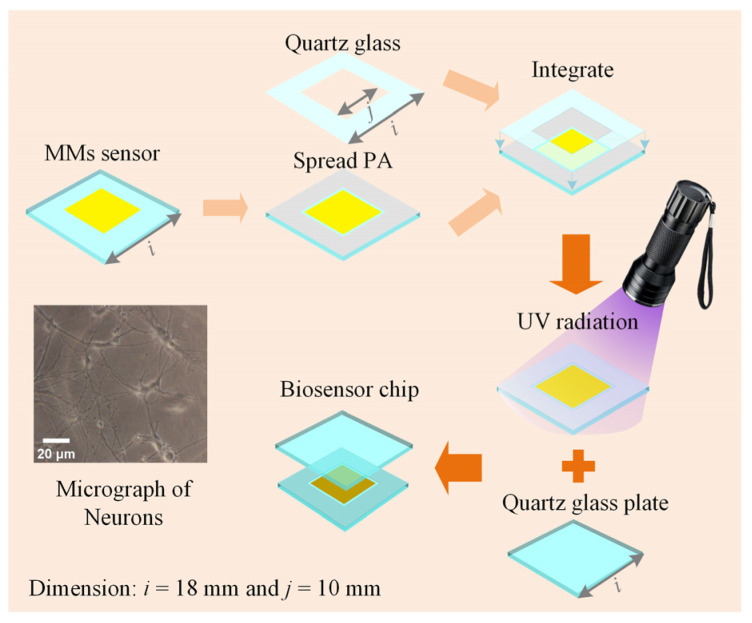
Schematic diagram of the biosensor chip and the micrograph of neurons.

The biosensor chip is essential for maintaining cell viability in THz biosensor detection. The chip consists of a high-sensitivity MMs sensor and two pieces of double-sided polished quartz glass (JGS1, Lianyungang Juxing Quartz Technology Co., Ltd., Lianyungang, China), as shown in Figure 3. Firstly, the sensor is coated with photosensitive adhesive (PA, ergo 8500, ergo company, Wetzikon, Switzerland) 4 mm from the edge. The sensor has 100 × 100 unit cells forming an 8 mm × 8 mm metal array. Then, a quartz glass of the same size as the sensor but with a different thickness is integrated. The quartz glass has an ultra-thin thickness of 50 μm with a 10 mm × 10 mm square hole. After that, the integrated structure is exposed to ultraviolet (UV) radiation to form a solid sample groove. Finally, the sample groove is positioned horizontally, and the solution is injected into it, after which the glass plate is placed over the sample groove. The sample groove and glass plate tightly adhere through atmospheric pressure and water surface adhesion, forming the biosensor chip. The biosensor chips are fixed by pressure applied by a 3D-printed assembly [15]. During testing, the sensor remains vertical and does not exhibit leakage. After testing, the biosensor chips can be detached using a wet razor blade and reused. Here, choosing ~50 μm for the ultra-thin internal cavity’s thickness of the sample cavity is a strategic decision. Generally, the cell has a diameter of around 10 μm–30 μm, as seen in the micrograph in the insets of Figure 3. The biosensor chip, with a depth of ~50 μm, can prevent adherent cells from being damaged by extrusion, weaken the strong water absorption of THz waves, and provide the liquid environment that is essential to mimic the in vivo cell state.

### 2.3. Sensitivity Analysis and Calculation

**Figure 4:**
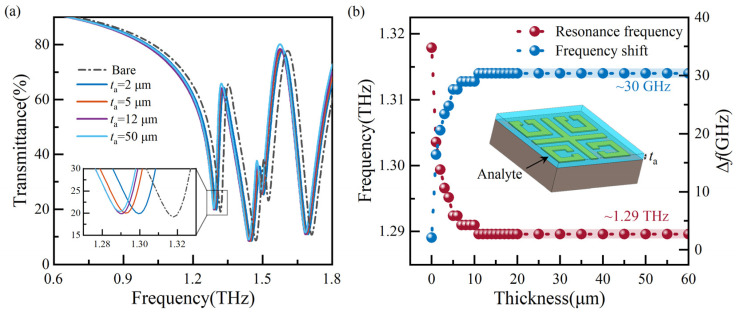
Analysis of transmission spectra with different thicknesses of the analyte: (**a**) a part of transmission spectra, and (**b**) resonance frequencies and ∆*f* extracted from simulated transmission spectra.

Sensitivity is analyzed to assess the sensor’s performance accurately. The analyte is placed on the top of the MMs sensor, as shown in Figure 4b insets. The thickness (*t*_a_) and dielectric constant (*ɛ*_a_) of the analyte affect the local electric field of the MMs sensor. In the case of determining the geometric dimensions of MMs, the effective dielectric constant (*ε*_eff_) is proportional to *ε*_a_. Any change in *ε*_a_ directly affects the sensor’s resonance frequency shift. *ɛ*_a_ can be obtained by the square of the refractive index (*n*_a_). Therefore, we focus on the influence of *t*_a_ and *n*_a_ on the sensor’s simulated transmission spectrum of the sensor.

When identifying biomolecules, the analyte has a specific thickness. Different dielectric constants identify the substance based on different transmission spectra. Therefore, it is necessary to explore the optimal thickness of the analyte. At *n*_a_ of 1.1, we simulated the transmission spectra for analytes with thicknesses ranging from 1 μm to 20 μm at one μm intervals and 20 μm to 60 μm at five μm intervals. For improved readability, Figure 4a shows a portion of transmission spectra, while Figure 4b displays the resonance frequency and frequency shift (∆*f*) extracted from the simulated transmission spectra. The increase in frequency shift gradually decreases with the increase in thickness from 1 μm to 11 μm, and similar resonance frequencies are found at thicknesses of 11 μm and 12 μm. With the increase in *t*_a_, the resonance frequency and ∆*f* level off at around 1.29 THz and 30 GHz, respectively. The sensor maintains optimal detection sensitivity when the analyte thickness is 50 μm. Here, the frequency shift ∆*f* denotes the frequency deviation corresponding to the resonance frequency compared to a bare state.

**Figure 5:**
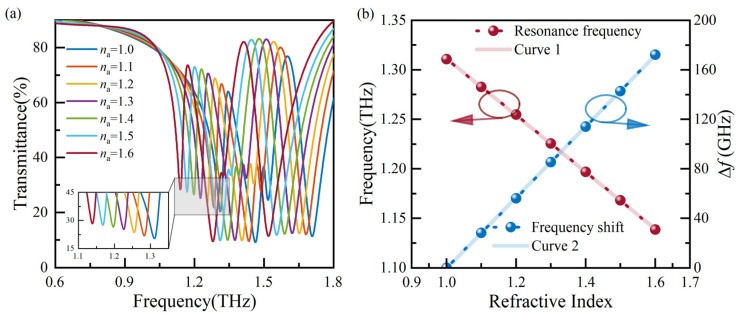
Analysis of transmission spectra with various analyte refractive indexes: (**a**) the transmission spectra, and (**b**) resonance frequencies and ∆*f* extracted from (**a**).

Subsequently, the influence of the *n*_a_ on the sensor’s sensitivity is analyzed. For the analyte’s thickness of 50 μm, a simulation is conducted with *n*_a_ ranging from 1.0 to 1.6, resulting in the transmission spectra shown in Figure 5a. As *n*_a_ increases, a redshift in the resonance frequency is observed. The resonance frequency and ∆*f*, corresponding to the refractive index, are extracted and plotted in Figure 5b. Linear fitting curves, denoted as Curve 1 and Curve 2, are obtained by performing linear regression analysis on the resonance frequency and ∆*f*, respectively. Curve 1 is described by the equation *f*(*n*_a_) = −0.287 × *n*_a_ + 1.599, and the theoretical sensitivity (*S*) is 0.287 THz/RIU.

## 3. Experimental Method

### 3.1. Experiment Setup

**Figure 6:**
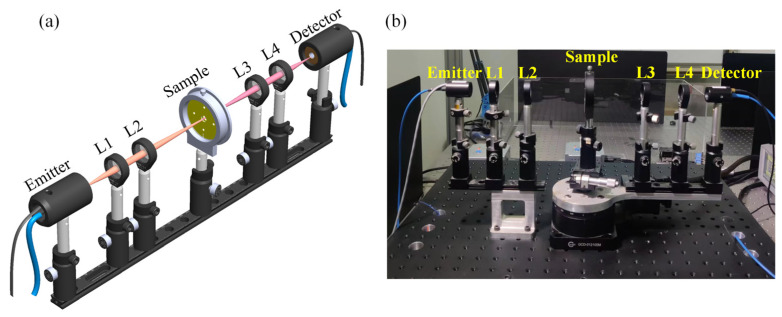
(**a**) Schematic diagram and (**b**) photograph of THz-TDS.

As shown in Figure 6, THz-TDS is used to analyze adherent cells in a biosensor chip with a typical spectral resolution of ~30 GHz [16,17]. The schematic diagram shows two photoconductive antennas (PCA), one emitter and the other detector, four lenses (L1, L2, L3, and L4), and a sample. A femtosecond laser (FemtoFErb FD 6.5, Toptica company, Gräfelfing, Germany) with a wavelength of 1550 nm, a power of 80 mW, a pulse duration of 60 fs, and a repetition rate of 100 MHz [18] drives the PCA, which generates photogenerated carriers that accelerate under the bias voltage and radiate electromagnetic waves in the THz band [19]. By using lens 1 (L1; focal length f1 = 50 mm), the divergent horizontally polarized THz beam is converted into a collimated beam and then focused on the sample through lens 2 (L2, focal length f2 = 100 mm) to obtain a beam with sample information. Subsequently, the beam becomes a collimated beam through lens 3 (L3; focal length f3 = 100 mm) and then passes through lens 4 (L4; focal length f4 = 50 mm) to the detector antenna. Finally, by scanning the delay line and using phase-lock amplification technology, the detection antenna transforms the THz beam into a current signal, realizing the detection of the terahertz electric field vector.

### 3.2. Culture of Adherent Cells

Glioma cells and neurons are cultured on a quartz glass plate using the adherent cell culturing method.

Glioma cells are obtained from the U87 cell line (RRID: CVCL_0022). They are cultured at 37 °C, with 5% CO_2_ in a culture solution consisting of a 9:1 mixture of Dulbecco’s Modified Eagle’s medium (DMEM High Glucose) and fetal bovine serum (FBS). Detailed parameters of chemical reagents are shown in Table 1. Once the cell density reaches a specified value, the culture medium is removed, and the cells are washed twice with phosphate-buffered saline (PBS). Then, 0.25% trypsin is added to digest cells, and the cells are transferred into a centrifuge tube and centrifuged at 1000 rpm for 5 min. After adding the resuspension cells with an adequate volume of liquid, a cell counting plate measures the cell density, aiming for 50,000 cells per unit. Next, the quartz glass substrates are placed in six-well culture plates, and 2 mL of culture medium is added and maintained in a 37 °C incubator. Finally, the cell suspension is added to the six-well culture plate with gentle agitation and shaking to ensure an even distribution of cells. The plates are returned to the 37 °C incubator to obtain the adherent cell on the surface of quartz glass.

Similarly, the adherent culture of neurons is cultured. Cortical neurons are obtained from neonatal mice (C57BL/6J, SPF biotech, Beijing, China) at the age of 0 days and are cultured with a starting population of 50,000 basal cells. The culture medium consists of 90% neurobasal-a medium and 10% FBS. The cells are carefully deposited onto the quartz glass substrates pre-coated with Poly-D-Lysine (PDL) and placed in a six-well culture plate. Each well in the culture plate has an area of 9.62 cm^2^. This culture is maintained in a 37 °C cell incubator with a 5% CO_2_ atmosphere. After the maturation period of 14 days, the experiments are undertaken with the cultured cells.

After the cells are attached to the quartz glass, the differentiation experiment is carried out between neurons and glioma cells. In addition, to test the apoptosis of glioma cells, we divided the remaining glioma cells into four groups. The glioma chemotherapeutic drug temozolomide (TMZ) is added to the cultures and incubated for various durations: 0 h, 24 h, 48 h, and 72 h, respectively. Apoptosis of the glioma cells is then assessed using THz technology.

### 3.3. Sample Testing Process

**Figure 7:**
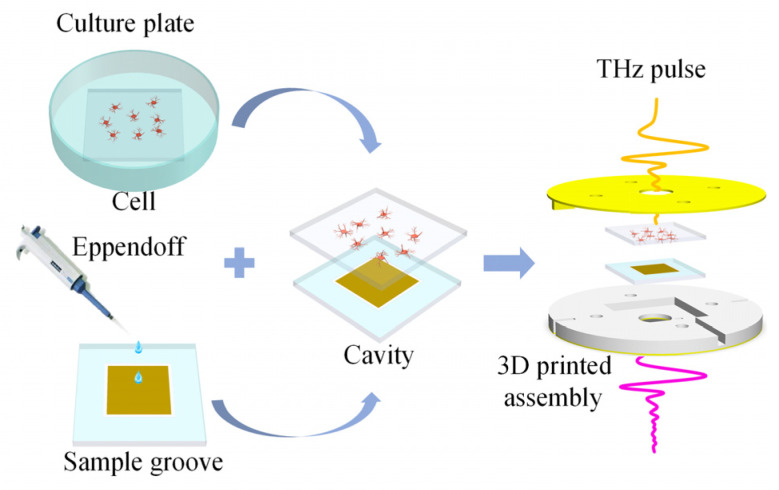
The process of sample testing.

The quartz glass plates with adherent cells are taken out of the six-well culture plates and washed twice in PBS to remove any excess organic matter that might affect the test results. The cleaned sample groove is positioned horizontally, and PBS is added to the sample groove with the MMs sensor to maintain the liquid environment required for cell viability. The quartz glass plates with the adherent cells are placed on top of the liquid sample groove, with the side of the adherent cells facing the liquid groove. Through the combined effects of atmospheric pressure and water surface adhesion, the glasses with liquid are tightly attached to form an ultra-thin liquid cavity [15]. This method can combine a high-sensitivity sensor with adherent cells in a liquid environment, offering numerous advantages. It enhances sensitivity, weakens the water’s strong absorption of THz waves, ensures uniform sample thickness, and closely mimics the in vivo state of the measured cells. Next, the chip with living cells is placed in a 3D-printed assembly to apply additional pressure for enhanced chip stability, followed by mounting this assembly onto an optical bracket (LM2, THORLABS, Newtown, America) used for THz-TDS. The steps for assembling the sample are illustrated in Figure 7. Finally, the obtained time-domain data are transformed by Fourier transform to obtain the frequency domain signal, and the frequency signal with the sample information is normalized by the reference signal to obtain the sample transmission spectrum. The reference chip consisted of three pieces of double-sided polished quartz glass, and unlike the biosensor chip, the metamaterial structure is replaced with a glass plate. The reference signal is obtained by the reference chip with PBS.

## 4. Measurement and Analysis

GBM is a highly malignant brain tumor with a poor prognosis. Therefore, early detection and effective treatment of GBM are crucial. Specifically, the initial experiments for early detection involve distinguishing glioma cells from Neurons. The usual treatment for GBM is to remove as much of the tumor as possible with surgery, then use radiation therapy along with anti-cancer drugs (such as TMZ) and chemotherapy afterward. Therefore, detecting the effect of TMZ on inducing apoptosis is essential for enhancing the treatment outcome.

### 4.1. Detecting Apoptosis in Glioma Cells

To assess the effects of TMZ on the apoptosis of glioma cells, the cells are cultured on the surface of quartz glasses in six-well culture plates at an initial concentration of 5200 cells/cm^2^. After a 24 h incubation, TMZ is added to the cultures and incubated for different durations: 0 h, 24 h, 48 h, and 72 h. Then, the commercial method (CCK-8) and the THz biosensor chip are used to measure cells’ apoptosis at different drug times.

**Figure 8:**
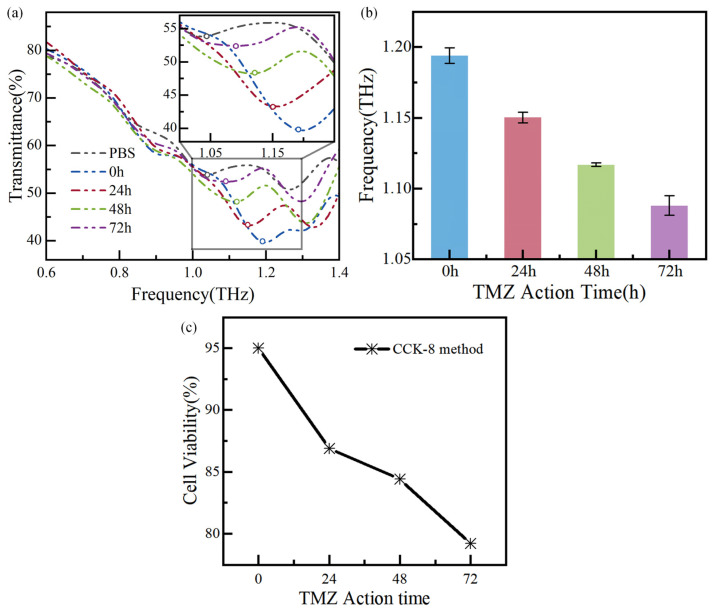
Measurement results of glioma cells with TMZ action time: (**a**) the transmission spectra of the biosensor chip, (**b**) error bars of the resonance frequency of the biosensor, and (**c**) the cell viability of the CCK-8.

Glioma cells with the TMZ action time of 0 h, 24 h, 48 h, and 72 h are measured successively, and the measurement results are shown in Figure 8a,b. The circle maker corresponds to the local minima in Figure 8a. The performance of biosensors is usually evaluated by analyzing the resonance frequency shift [20]. As shown in Figure 8a, the resonance frequency of the PBS solution is 1.04 THz, with a frequency shift of 270 GHz. Additionally, the resonance frequencies change from 1.19 THz, 1.15 THz, 1.12 THz, and 1.09 THz, respectively, as the drug action time is increased from 0 h to 72 h, and the corresponding frequency shifts are 120 GHz, 160 GHz, 190 GHz, and 220 GHz, respectively. The relative frequency shift is for the biosensor’s bare state (~1.31 THz). The resonance frequency shift of adjacent curves of different apoptosis times is at least 30 GHz. The resonance frequencies are measured multiple times and plotted in Figure 8b, showing the measurement error below 0.64%.

In parallel, the apoptosis of glioma cells is also detected using the CCK-8 method, as shown in Figure 8c. For this, the optical density (OD) at 450 nm is measured using a UV–visible spectrophotometer one hour after incubation with CCK-8 method. The following equation calculates the cell viability:

Cell viability = [(*A*_s_ − *A*_b_)/(*A*_c_ − *A*_b_)] × 100% (1)

where *A*_s_ represents the absorbance of the experimental well, *A*_c_ represents the absorbance of the control well, and *A*_b_ represents the absorbance of the blank well.

According to the commercial method, the cell concentration can be calculated, as shown in Table 2. The method has demonstrated that as the TMZ action time increases from 0 to 72 h, the number of living glioma cells decreases while the number of disabled cells increases. Necrosed and ruptured cells are washed away with PBS in the sample testing process, leading to an increase in PBS content in the tested chip. Since the dielectric constant of water solutions and culture media is higher than that of living tumor cells [21,22], the *ε*_a_ increases, resulting in a red shift in the resonance frequency. Additionally, the literature [22] confirms a decrease in cell viability and an increase in the dielectric constant, consistent with the test results from the biosensor. In summary, the designed THz biosensor chip has significant potential for cancer detection, as the frequency shift it detects agrees with the commercial method measurement. This study provides a new, fast, low-cost, and high-sensitivity way to realize biosensing from a physical perspective.

### 4.2. Identification of Different Types of Cells

**Figure 9:**
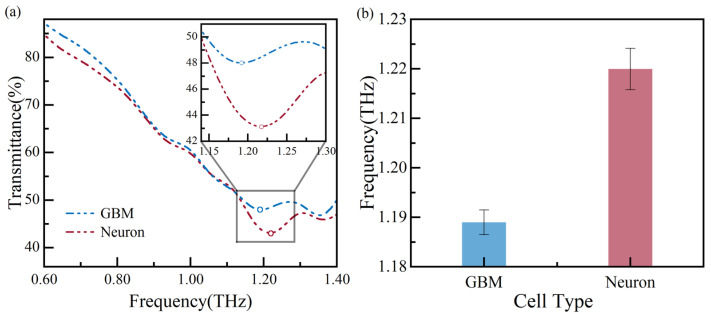
Measurement results of different types of cells: (**a**) the transmission spectra and (**b**) error bars of the resonance frequency.

Using THz spectroscopy, glioma cells and neurons are cultured to verify the biosensor’s ability to distinguish between different types of cells at a concentration of ~5200 cells/cm^2^. The measurement results are shown in Figure 9, and the circle maker corresponds to the local minima in Figure 9a. The figure displays the frequency shift between glioma cells and neurons. The glioma cell’s and neuron’s resonance frequency shifts to 1.19 THz and 1.22 THz, respectively, with a resonance frequency difference of ~30 GHz. The resonance frequencies are measured multiple times and plotted in Figure 9b, showing the measurement error is less than 0.35%. The resonance frequency of glioma cells is lower than that of neurons, indicating that glioma cells have a significantly higher refractive index than neurons. This finding is consistent with previous reports [23,24,25,26,27]. Based on the above analysis of results, our measured results suggest that the glioma cells and neurons can be distinguished at a concentration of ~5200 cells/cm^2^.

### 4.3. Performance Comparison

A comparison between the presented approach and the other sensors is presented in Table 3, where *f*_0_ represents the resonance frequency of the bare sensor and *C*_cell_ denotes the concentration of the measured cell. Based on this table, different biosensing approaches exhibit diverse sensitivities (*S*) and resonance operational frequencies. Thus, the normalized sensitivity *S_f_*_0_ is considered to ensure a fair comparison, where *S_f_*_0_ is *S* normalized to *f*_0_ [28]. As Table 3 shows, the proposed sensor shows a higher sensitivity than the other sensors. The samples measured in the literature [9,11,14,29] are either dry or partially hydrated specimens that do not reflect in vivo biological status. Our method can weaken the strong water absorption by THz waves without compromising the cell activity during the test. In summary, this work can achieve cancer detection with higher sensitivity in an in vivo-like cell state at a low cell concentration.

## 5. Conclusions

In this study, a detection method for cells that mimic the in vivo state by a biosensor chip is presented. The high-sensitivity biosensor is simulated by the various analytes having different refractive indexes and thicknesses. The theoretical sensitivity is calculated as 0.287 THz/RIU at the analyte thickness of 50 μm. Using the biosensor chip, the apoptosis of glioma cells is evaluated under 500 μM TMZ of different action times. As the drug action time increases from 0 to 72 h, the resonance frequencies vary from 1.19 THz, 1.15 THz, 1.12 THz, and 1.09 THz, respectively. The resonance frequency shift trend of the biosensor method agrees with that of the commercial method. Furthermore, the ability of the biosensor to distinguish between neurons and glioma cells is verified. At a concentration of ~5200 cells/cm^2^, glioma cells and neurons are distinguished with a resonance frequency difference of ~30 GHz. Based on experiments, the biosensor method can detect the different types of adherent cells and the apoptosis of glioma cells at an in vivo-like condition. The proposed method has advantages such as high-sensitivity detection, closing similarity to the in vivo cell state, weakening the strong absorption by water, ensuring sample thickness consistency, reducing the number of MMs chips required for multiple data sets, preventing MMs structure damage during cleaning, and cultivating adherent cells uniformly on the metal pattern of MMs. In conclusion, this study can provide a new THz high-sensitivity detection method at the in vivo-like cell state. It has great potential in detecting GBM and provides a better alternate method for bioactive cell detection.

## Figures and Tables

**Table 1:** Parameters of chemical reagents.

Chemical Reagent	Source	Identifier
DEME High Glucose	Biosharp	Cat. No: BL304A
FBS	Gibco	Cat. No: 10099141C
Trypsin	Biosharp	Cat. No: BL512A
PBS	Sigma	Cat. No: D8537
Neurobasal-A medium	Gibco	Cat. No: 10888-022
PDL	Sigma	Cat. No: P4707
Temozolomide	Sigma	Cat. No: T2577
Cell Counting Kit-8	MedChemExpress	Cat. No: HY-K0301

**Table 2:** The cell concentration of measurement results with the CCK-8 method.

TMZ Action Time (h)	Cell Viability (%)	Cell Concentration (Cells/cm^2^)
0	~95.0	~4938
24	~86.9	~4516
48	~84.4	~4386
72	~79.2	~4116

**Table 3:** Comparison with other methods.

Ref.	*f*_0_	*S*	*S_f_*_0_	*C*_cell_	Analyte
[9]	0.850 THz	0.074 THz/RIU	8.7%	-	Vitro tissues
[11]	0.850 THz	0.182 THz/RIU	21.4%	2 × 10^6^ cells/mL	Dried adherent cells
[14]	0.777 THz	0.118 THz/RIU	15.2%	1 × 10^5^ cells/cm^2^	Adherent cells
[29]	1.180 THz	0.249 THz/RIU	21.1%	0.5 × 10^5^ cells/mL	Adherent cells
This work	1.320 THz	0.287 THz/RIU	21.7%	5200 cells/cm^2^	In vivo-like cells

## Data Availability

The data presented in this study are available on request from the first author.
